# Virtual agents and risk-taking behavior in adolescence: the twofold nature of nudging

**DOI:** 10.1038/s41598-023-38399-w

**Published:** 2023-07-11

**Authors:** Cinzia Di Dio, Federico Manzi, Laura Miraglia, Michaela Gummerum, Simone Bigozzi, Davide Massaro, Antonella Marchetti

**Affiliations:** 1 Research Unit On Theory of Mind, Department of Psychology, Università Cattolica del Sacro Cuore, Milan, Italy; 2 Research Unit On Robopsychology in the Lifespan, Department of Psychology, Università Cattolica del Sacro Cuore, Milan, Italy; 3 Department of Psychology, University of Warwick, Coventry, UK; 4 QuestIT s.r.l., Siena, Italy

**Keywords:** Psychology, Human behaviour, Risk factors, Social evolution, Cultural evolution

## Abstract

Peer pressure can influence risk-taking behavior and it is particularly felt during adolescence. With artificial intelligence (AI) increasingly present in a range of everyday human contexts, including virtual environments, it is important to examine whether AI can have an impact on human’s decision making processes and behavior. By using the balloon analogue risk task (BART) evaluating propensity to take risk, in this study 113 adolescents' risk-taking behavior was measured when playing alone and in the presence of either a robot avatar or human avatar. In the avatar conditions, participants performed the BART while the avatars either (1) verbally incited risk-taking or (2) discouraged risk-taking (experimental tasks). Risk-taking behavior in the BART was assessed in terms of total number of pumps, gain and explosions. Tendency to impulsivity was also evaluated, as well as the effects of age and gender on risky behavior. The main finding showed a significant effect of both avatars on risk-taking tendency, with riskier behavior during incitement than discouragement conditions, the latter being also substantially different from the playing-alone condition. The results of this study open up new questions in a very sensitive and timely topic and offer various insights into the effect of nudging on adolescents’ behavior in virtual contexts.

## Introduction

For the digital native generations, the internet is an environment where they spend a significant part of their daily life. Nowadays, activities such as education, medical care, shopping, exhibitions, and tourism have moved from being only offline to also virtual^1^, constantly exposing people to Artificial Intelligence (AI). In the metaverse, for example, people use body avatars to interact realistically (i.e., speech, facial expressions, and body language) with each other concretely experiencing the Onlife (i.e., a physical and digital hybrid life^2^), and avatars will also be increasingly used by government institutions and companies to interact with people^3^.

Avatars can potentially exert social influence on human behaviors by shaping individual decision-making. While the cyberspace provides opportunities to enhance and broaden life experiences and represents a venue for leisure and educational activities among young people, the increasing permeation of the internet into their lives exposes them to information with questionable credibility, ideas that potentially undermine positive behaviors, and messages that are intended to manipulate their actions or beliefs^4,5^. On the one hand, the cyberspace is increasingly a point of social contact for adolescents who may prefer the perceived anonymity of internet relationships. Thus, online interactions with virtual entities, i.e., avatars, may create a sense of comfort and be seen as less intimidating for young people, leading to reduced inhibitions and decreased fear of social judgment. On the other hand, as youth spend more time online, they face an increased risk of being exposed to factors that diminish their ability to self-regulate and increase their tendency to engage in impulsive actions^6^. The absence of emotional feedback and detachment from the real-world consequences of their actions, combined with a reduced fear of punishment, potentially contribute to poor decision-making^7^ and lead to a greater likelihood of engaging in reckless behaviors^5,8^. This effect becomes relevant when virtual agents actively shape adolescents’ attitudes and actions in the online realm through strategies like gamification, rewards, or personalized recommendations. The sensitive transitional period of adolescence makes young people more susceptible to these strategies due to the peak of risk-taking behavior specific to this age^9,10^. Recent findings from the field of neuroscience have provided important insight into how changes in neuroanatomic and neural activity through adolescence contribute to a potential rise in risk-taking behavior^11^. Developmentally, adolescence is frequently described as a phase marked by increased risk-taking tendencies and exceptionally vulnerable to risky behavior—particularly in early adolescence^12–14^, including poor decision-making, in which a youth’s sense of invulnerability results in a failure to consider risks^15^.

In live interactions, current literature consistently suggests that, during adolescence, peer relationships play an increasingly significant role, providing a crucial milieu for young people to test their social skills. Adolescents tend to spend more time with peers and attach great importance to their expectations and opinions^16^. Peer influence may represent a risk factor, reinforcing the development of unsafe behaviors such as substance abuse, reckless driving, and criminal activities^17–20^. However, it is important to recognize the bidirectional nature of peer pressure outcomes. Peer pressure can also act as a protective factor, promoting cautious behavior^21^. For instance, a deviant adolescent may gradually adopt less deviant behavior influenced by a nondeviant peer^22^. In the virtual world, avatars can interact, suggest, and even simulate peer-like relationships^23^, with the potential to shape adolescents’ actions in a manner comparable to peer influence and, although a growing body of studies has proved the influence of (offline) peer pressure on adolescents’ behaviors—particularly focusing on risk-taking^24–28^, research on the impacts of avatars on behavior remains still sparse. Additionally, to date, research has widely focused on the potential negative effect that technologies can exert on adolescents (e.g., game addiction, fake news)^29,30^, while rarely addressing its positive influence on individuals’ behaviors. The current research addresses both these questions.

Avatars can take any form, more or less anthropomorphic. In recent years, artificial intelligence is increasingly being associated with the use of humanoid robots in interactions with people of various ages, from toddlers to the elderly (e.g., Di Dio et al.^31^; Manzi et al.^32^; for a review, see Marchetti et al.^33^). Additionally, some studies have also addressed peer-pressure using humanoid robots as activity partners. Drawing on Asch’s conformity experiment^34^, which shows that people are prone to adjust their opinions to match those of group members even when they believe the unanimous majority response is wrong, research^35–42^ has examined whether humans would conform to a group of robots with unanimous but erroneous judgments. Brandstetter and colleagues^35^ failed to observe conformity with a group of robots; conversely, other studies have found that robots can cause informational and normative conformity^37,38^ in children^40^ and adults^36^, thus leaving the issue of AI-influence on human behavior still open to question. Additionally, building on the Balloon Analogue Risk Task (BART)^9,43,44^, Hanoch and colleagues^45^ made an attempt to explore the nudging effect of robots on risk-taking behavior in undergraduate students. The BART is a digital game in which participants have to inflate a virtual balloon to get scores. The more the balloon is inflated, the higher the score obtained. However, the balloon can explode at any time on a probabilistic base. The participant’s risk-taking is weighted against the probability of gain. The participants completed the BART alone, in the presence of a silent robot, and in the presence of a robot that provided explicit statements encouraging risk-taking. Participants who completed the BART in the risk-incitement robot condition exhibited higher risk-taking behavior compared to other groups, thus lending support to data suggesting a significant effect of robot’s influence on human behavior.

Drawing inspiration from Hanoch and colleagues’ experimental design^45^ and building upon the Balloon Analogue Risk Task (BART)^9,43,44^, the present study aimed to empirically explore whether avatars can influence adolescents’ risk-taking behavior, either by encouraging or discouraging it. In light of the above studies and evidence, we also took the opportunity to compare adolescents' behavior in response to more or less anthropomorphic avatars, thus endowing the avatars with a robotic or human appearance. Consequently, participants underwent the BART both individually to assess their risk-taking tendencies when playing alone and in the presence of either a robot or a human avatar. In the conditions with the avatars (experimental conditions), the virtual entities were presented on the screen of the computer used to play the BART game and engaged with the participants while playing the BART. Specifically, the virtual agents were set to either verbally incite or discourage risk-taking behavior by delivering either encouragement statements (e.g., don’t be afraid, don’t stop) or dampening statements (e.g., be careful, slow down). To evaluate the effects of the incitement and discouragement conditions on the participants’ behavior, BART scores were computed in terms of total number of key presses (pumps), total gain (gain), and total explosions (explosions). Consistent with the results of the original work^45^, the incitement condition was expected to nudge participants’ behavior by increasing risk-taking and, by contrast, discouragement to positively nudge behavior thus acting as a protective factor against risk-taking. Confirming the experimental hypotheses would support, from an empirical point of view, the need to carefully evaluate the inclusion of artificial agents in virtual realms when teens are operating online. This would be most important if a risk-encouraging effect were found. Conversely, a behavioral mitigation effect as a function of positive nudging by the virtual agent (i.e., discouragement condition), would support, for example, the implementation of prevention programs that make use of avatars to promote positive behaviors and discourage negative behaviors in, for example, anti-smoking, -vandalism, and -bullying campaigns^46^. In this respect, applications can be limitless.

Finally, individual inclination to be influenced by peers in risky behaviors has been associated with demographic variables including gender, i.e., males are more susceptible to peer influences than females (for a review, see McCoy et al.^34^) and age, i.e., peer pressure increases during early adolescence, peaks around the age of 14, and declines thereafter^47,48^. Therefore, age and gender were here assessed as predictors of the participants’ behavior on the BART task. Alongside the demographic variables, the tendency to impulsivity—defined as the disposition to behave in a precipitous manner and without adequate reflection^36^—was also assessed.

## Results

### Checking extremes

A visual inspection of the boxplots representing the dependent variables (pumps, gain, explosions) in the playing-alone and avatar conditions, separately for the incitement and discouragement modality, revealed the consistent presence of two cases identified as extremes and/or outliers in at least 50% of the conditions, particularly during incitement. These cases were thus removed from the analyses. The final sample of 113 participants showed skewness indexes within acceptable ranges (+ − 2) in all conditions [incitement range = − 0.33–1.65 (ES = 0.316); discouragement range = 0.09–1.03(ES = 0.319)]. See Table 1 for mean scores and asymmetry indexes.

**Table 1:** Mean, standard deviation, minimum and maximum values, asymmetry (skewness) indexes for total number of pumps (pumps), total gain (gain) and total explosions (explosions) in the playing-alone (P-A) and avatars (AV) conditions during incitement and discouragement modalities.

	Min	Max	Mean	Stand dev	Asymmetry	
					Stat	Stand error
Incitement, N = 57						
Pumps P-A	325	1808	1203.86	374.04	− 0.59	0.316
Pumps AV	409	2128	1201.81	338.70	0.12	0.316
Gain P-A	9	1619	459.16	336.22	1.65	0.316
Gain AV	16	1145	441.42	262.94	1.20	0.316
Explosions P-A	2	29	12.74	5.53	0.81	0.316
Explosions AV	2	21	12.46	4.10	− 0.05	0.316
Discouragement, N = 56						
Pumps P-A	368	1977	1157.95	330.94	0.09	0.319
Pumps AV	290	1715	1041.59	367.72	− 0.09	0.319
Gain P-A	14	1140	404.32	276.99	1.03	0.319
Gain AV	29	890	302.96	202.11	1.02	0.319
Explosions P-A	2	21	11.50	4.48	0.30	0.319
Explosions AV	4	20	10.34	3.71	0.36	0.319

#### Descriptive analysis

As shown in Table 2, age and gender-based distribution of participants was similar across the experimental conditions, with a prevalence of males in each group.

**Table 2:** Participants’ distribution for gender and age (years) in the human avatar and robot avatar discouragement and incitement conditions (N = 113).

		n, mean age (Std err)	
		Male (N = 80)	Female (N = 33)
Human avatar	Discouragement	20, 17.25 (0.21)	8, 16.75 (0.16)
	Incitement	20, 16.89 (0.20)	9, 17.35 (0.20)
Robot avatar	Discouragement	20, 17.10 (0.19)	8, 17.13 (0.35)
	Incitement	20, 17.65 (0.27)	8, 17.25 (0.16)

#### Construct consistency checks

Positive correlations were found between the three BART variables as reported in Table 3, showing consistency in the construal of risk-taking within each played BART.

**Table 3:** Correlations between BART scores (pumps, gain, explosions) in the experimental conditions.

Playing alone	Pumps	Gain	Explosions
Pumps	1	0.77**	0.71**
Gain	–	1	0.92**
Explosions	–	–	1
Playing with avatar			
Pumps	1	0.85**	0.76**
Gain	–	1	0.90**
Explosions	–	–	1

** Correlations is significant at the level 0.01 (one tailed).

Also, positive correlations were found between the BART variables evaluated when the participants played alone and the same BART indices when playing with the avatars. That is, independent of incitement or discouragement modalities, people that displayed greater tendency to risk when playing alone, did so also in the conditions where the avatars either incited or discouraged risk-taking, thus supporting inter-subject consistency in risk-taking tendency. Statistics are presented in Table 4.

**Table 4:** Correlations between BART scores when playing alone (pumps, gain, explosions) and BART scores when playing with the avatar.

Type of agent	Modality		Playing alone		
			Pumps	Gain	Explosions
Robot avatar	Discouragement	Pumps	0.57**	–	–
		Gain	–	0.65**	–
		Explosions	–	–	0.47*
	Incitement	Pumps	.71**	–	–
		Gain	–	.68**	–
		Explosions	–	–	.69**
Human avatar	Discouragement	Pumps	0.56**	–	–
		Gain	–	0.70**	–
		Explosions	–	–	0.82**
	Incitement	Pumps	0.31*	–	–
		Gain	–	0.36**	–
		Explosions	–	–	0.61**

**The correlation is significant at level 0.01 (one tailed).

*The correlation is significant at level 0.05 (one tailed).

Three independent general linear models (GLM) comparing the BART scores between conditions (playing-alone, avatar condition), modality (incitement, discouragement) and type of agent (human, robot avatar), were carried out each with the following dependent variables: total number of presses (pumps), total final score (gain), and total number of explosions (explosions). Gender was also included in the model as a between-subjects factor.

**Figure 1:**
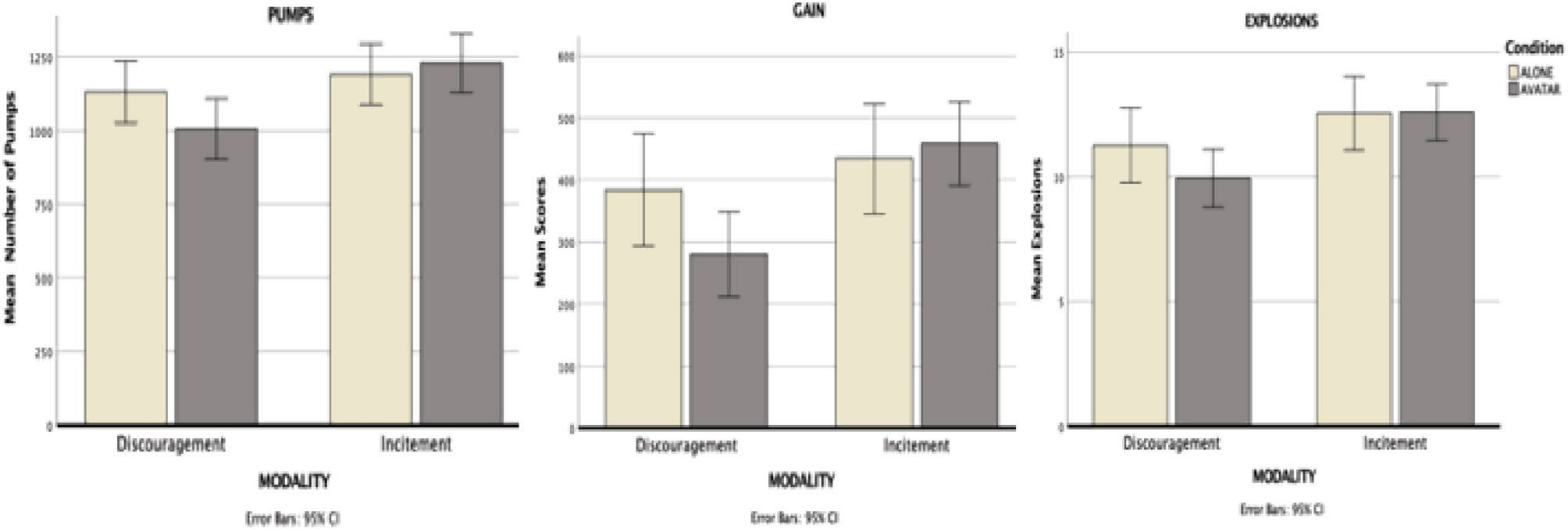
The graphs represent the interaction between playing-alone and playing-with the avatar conditions in the BART task for the incitement and discouragement modalities. The dependent variables are the mean scores for (from left to right) pumps, gain and explosions.

The three models showed quite consistent findings across the dependent measures. The main results showed a main effect of modality for all three variables (pumps, *F*_*(105)*_ = 4.77*, p* < *0.05, partial-η*^*2*^ = 0.04*, δ* = 0.58; gain, *F*_*(1, 105)*_ = 5.09*, p* < 0.05, *partial-η*^*2*^ = 0.05*, δ* = 0.61; explosions, *F*_*(1, 105)*_ = 5.28*, p* < 0.05, *partial-η*^*2*^ = 0.05*, δ* = 0.62), indicating significantly lower scores in the discouragement than incitement conditions. Additionally, for pumps and gains, a significant interaction was found between condition and modality (pumps, *F*_*(105)*_ = 5.68*, p* < *0.01, partial-η*^*2*^ = 0.06*, δ* = 0.70; gain, *F*_*(105)*_ = 6.26*, p* < *0.01, partial-η2* = 0.05*, δ* = 0.66), showing that number of pumps was substantially lower during the discouragement condition than when playing alone (pumps: *M*diff = 124.05, *SE* = 48.28, *p* < 0.01, gain: *M*diff = 103.37, *SE* = 36.34, *p* < 0.01). Although this interaction just failed to reach significance for explosions, the difference between discouragement and playing-alone condition was still preserved (*M*diff = 1.32, *SE* = 0.58, *p* = 0.02, Bonferroni corrected). The interactions are plotted in Fig. 1.

No differences were either found between playing-alone and incitement condition, between type of agent (HA, RA), or gender for any of the dependent variables (pumps, gain, explosions; ns).

### Regression analyses

Three independent regression analyses evaluated the predictive effect of the BART variables. More specifically, *pumps, gain, and explosions* were regressed separately on the independent variables *modality* (incitement, discouragement—dichotomously defined 0 = discouragement, 1 = incitement) and *type of avatar* (HA, RA), controlling for participants’ age and gender (model 1), impulsive behavior (BIS11; model 2), and BART scores when playing alone (model 3).

Summarizing our findings, there was no significant effects of age and gender on any of the BART variables (Model 1; *pumps*: *F*_2,110_ = 0.04, ns, *R*^*2*^ = 0.001, *R*^*2*^_*adjusted*_ = − 0.02; *gain*: *F*_2,110_ = 1.06, ns, *R*^*2*^ = 0.02, *R*^*2*^_adjusted_ = 0.001; *explosions*: *F*_2,110_ = 0.37, ns, *R*^*2*^ = 0.007, *R*^*2*^_*adjusted*_ = − 0.011). Furthermore, no significant predictive effect of impulsivity was found on pumps, gain or explosions (Model 2; *pumps*: *F*_5,107_ = 0.79, ns, *R*^*2*^ = 0.04, *R*^*2*^_*adjusted*_ = − 0.01; *gain*: *F*_5,107_ = 1.10, ns, *R*^*2*^ = 0.05, *R*^*2*^_adjusted_ = 0.005; *explosions*: *F*_5,107_ = 0.78, ns, *R*^*2*^ = 0.05, *R*^*2*^_*adjusted*_ = − 0.01). On the other hand, a substantial effect of BART scores evaluated when playing alone was found on all BART variables (Model 3; *pumps*: *F*_6,106_ = 8.80, *p* < 0.001, *R*^*2*^ = 0.33, *R*^*2*^_*adjusted*_ = 0.30; *gain*: *F*_6,106_ = 10.73, *p* < 0.001, *R*^*2*^ = 0.42, *R*^*2*^_*adjusted*_ = 0.38 s *R*^*2*^ = 0.45, *R*^*2*^_*adjusted*_ = 0.41).

Finally, after controlling for all the above variables, the target predictor *Modality* (0 = discouragement; 1 = incitement)—but not *Type of Avatar* (0 = robot; 1 = human)—showed to significantly predict all BART scores, with incitement being associated with greater tendency to risk-taking (Model 4: *pumps*: *F*_6,104_ = 7.63, *p* < 0.001, *R*^*2*^ = 0.37, *R*^*2*^_*adjusted*_ = 0.32, Durbin-Watson = 1.8; *gain*: *F*_8,104_ = 9.54, *p* < 0.001, *R*^*2*^ = 0.42, *R*^*2*^_*adjusted*_ = 0.38, Durbin -Watson = 2.0; *explosions*: *F*_8,104_ = 11.97, *p* < 0.001, *R*^*2*^ = 0.48, *R*^*2*^_*adjusted*_ = 0.44, Durbin Watson = 1.83). By contrast, discouragement significantly predicted lower risk-taking as evaluated after modelling the same variables as above described, thought reversing the dichotomy index, where 0 represented incitement and 1 discouragement. Table 5 summarizes the statistical details and Supplemental Table 4 the complete regression model.

**Table 5:** Regression analysis for variables predicting performance at the BART task played in the experimental condition (with avatar).

Variable	Pumps			Gain			Explosions		
	B	SE(B)	*β*	B	SE(B)	*β*	B	SE(B)	*β*
Age range	− 40.93	32.06	− 0.10	12.07	20.66	0.05	0.01	0.32	0.00
Gender	− 25.14	63.68	− 0.03	− 50.43	41.37	− 0.09	− 0.00	0.65	0.00
BIS11-Att	0.43	11.06	0.00	− 0.18	7.13	− 0.00	− 0.03	0.11	− 0.02
BIS11-Mot	5.55	7.17	0.07	− 1.58	4.68	− 0.03	− 0.08	0.07	− 0.09
BIS11-NonPl	0.10	6.49	0.00	5.91	4.19	0.11	0.08	0.07	0.09
Playing-alone	0.57	0.08	0.56	0.46	0.06	0.58	0.53	0.06	0.66
Type of agent	− 16.99	56.18	− 0.02	10.47	36.31	0.02	0.52	0.57	0.06
Modality	141.46	57.16	0.19	105.22	37.04	0.22	1.43	0.59	0.18

To summarize the main results, here are reported data for Model 4 encompassing all the variables for each regression. The full model is in Supplementary Material Table 1.

Predictor regression 1: pumps (N = 113).

Predictor regression 2: gain (N = 113).

Predictor regression 3: explosions (N = 113).

*p < 0.05.

**p < 0.01.

***p < 0.001.

## Discussion

Can avatars exert pressure to impact adolescents’ risk-taking behavior? Our results showed a substantial effect of both human and robot avatars’ influence on adolescents’ behavior, more evident in the discouragement condition. That is, participants’ risk-taking substantially decreased when the avatars verbally discouraged risk-taking compared to a condition in which participants played the BART alone. On the other hand, no significant differences were found between playing-alone condition and the incitement condition, although the latter showed greater scores compared to the discouragement condition. No differences were found between playing-alone conditions as well, thus indicating homogeneity of the participants’ behavior at start across all conditions for all measured indexes. Additionally, we found no effect of type of virtual agent (human or robot) on behavior, i.e., our research suggests that the effect of the virtual agent on behavior is independent of its level of anthropomorphism, as both human and robot avatars used in this study produced similar effects. Finally, data showed no effect of either age or gender on risk-taking.

The main finding of the present study suggests a significant positive nudging power of virtual avatars to influence individuals’ behavior in late adolescence, thus outlining a potential protective effect on online behavior. Previous studies investigating peer influence are aligned with the present findings, showing a tendency—specifically in late adolescence—to make less risky choices following other’s decisions than young adolescents^49^. Additionally, in an adapted version of the BART, participants were evaluated under the potential influence of peers’ cautious or risky behavior in situations where they were either informed or not about the level of risk featuring the game^50^. It was shown that peers’ cautious choices substantially decreased participants’ risk-taking in noninformed conditions. This situation mirrors the general context in which our participants played the BART, and the results reflect the significant effect of risk deterrence specifically found in the discouragement condition. In contrast peers’ risky choices in Osmont et al.^50^ increased—under peer pressure—adolescents’ risk-taking when risk was minimal.

These observations may partly explain why we found no differences in risk-taking between the incitement and the playing-alone conditions. Our incitement condition—totally uninformed in Osmont et al.^50^ terms—would dampen the effect of risk-taking under the influence of others, even when these others are virtual agents, as in our case. To this, we should also add that the playing-alone condition plausibly brought with it a task-familiarization effect that, in a sense, led our participants to explore the extent to which they could push themselves to take risks. While this condition duly reflects participants' idiosyncratic tendency to incur risk-taking, in a way it also encourages risk-taking through a natural process of exploration. Taken together, these reflections suggest that the avatars in our study exerted an effect on risk-taking during the incitement condition, keeping the level of risk rather high regardless of the levels of risk uncertainty that characterize BART.

The predictive effect of solicitation modality (incitement, discouragement) on all three BART variables found in the regression analyses supports the above suggestion by showing that—net of the familiarization effect in the playing-alone condition—avatar solicitations increased or decreased risk-taking depending on whether the participants played in the incitement or discouragement condition, respectively. This is further reinforced by the differences found between incitement and discouragement conditions for all three BART variables (pumps, gain, explosions). These results partially support previous findings^45^ where undergraduates played in vivo with a robot that verbally encouraged risky behavior at the BART. The authors found a significant increase in participants’ risk-taking under the influence of the robot’s solicitations with respect to those who played the BART alone or when the robot was present in a silent condition. Failure to find an actual difference between our playing-alone and incitement condition may refer partly to the explanations provided above and that do not fully apply to Hanoch et al.’s^45^ study, in which the playing-alone condition was kept in a between-subjects design. Also, the agent in Hanoch et al. was physically present, while our agents were virtual. Embodiment may have then further contributed to the difference found from our results^51,52^.

Demographic variables gender and age were further assessed in this study as they have been associated with individual inclination to risky behaviors in adolescence^47,53^. Neither age nor gender significantly predicted behavior at BART. In the present study, participants were equally distributed, although boys outnumbered girls in all conditions, thus possibly obscuring a potential gender effect. Future studies should take this variable into account to properly assess this possibility. Furthermore, our students ranged in age from 16 to 18 years and, thus, the lack of age differences in our study could be plausibly due to low variably in the age range. Additionally, previous studies have shown different tendencies in risk-taking behavior between early and late adolescence, with the latter group displaying safer behavior than younger teens. The study by Braams et al.^49^, for example, examined how social information influences risky and ambiguous choices in adolescence, using an economic decision-making approach. Participants chose between safer and riskier lotteries and received information about other participants' choice preferences. Results suggest that late adolescents were less likely to follow risky choices and more likely to follow safe choices than younger adolescents. As suggested by the authors, this tendency likely stems from social motivations and a desire to conform to safer norms in late adolescence, which may—at least partially—explain why our adolescents group showed significant less risky behavior when incited to do so. Also, Somerville et al.^54^ found a decreased tendency to take risk with increasing age. Our results thus extend the current literature by showing that protective actions can also be effective when exerted by virtual agents in online activities. Other socio-demographic variables could be considered—in addition to age and gender—to further enrich the topic of online nudging in adolescence. These possibilities have been outlined in the "future directions" section below.

Research also suggests the influence of socio-psychological factors such as personality traits—like sensation seeking^18,19^, and impulsivity ^46,55^—on risk-taking behavior in adolescents. In the present study, we evaluated impulsive behavior on three dimensions: motor-activation, attention, and lack-of-planning. None of the investigated dimensions significantly predicted risk-taking at the BART. Again, the failure to find a substantial effect of these variables could be due to the fact that our participants were toward late adolescence and, therefore, less inclined to seek strong sensations and less impulsive, especially when the outcome of one’s behavior cannot be predicted. This would be in line with the neurophysiological development of executive abilities, which reach maturation in late adolescence^48^.

In sum, this study shows that—regardless of their physical features (human or robotic)—avatars have an effect on risk-taking by acting on both discouragement (positive nudge) and incitement (negative nudge) of behavior. This finding is particularly informative for multiple activities that people can experience within virtual environments, as avatars will be increasingly present in supporting people’s decision-making, and thus influencing their choices. From an ethical perspective, it will be particularly important that these avatars—whose online presence is and will be increasingly prevalent and inevitable—are used with the purpose of reducing those behaviors that affect risky decision-making processes. In this respect, we explored the effect of avatars only in a risk-taking situation, but it will be very informative to assess their influence also in prosocial scenarios, i.e., positive behavior.

The encouraging message from our data—preliminary in nature—is that the nudging action of virtual agents can be bidirectional, thus correcting rather than reinforcing risky behavior. Knowing that there may be negative effects on behavior may help prevent risk factors, and knowing that the effect may act in a positive direction may help strengthen protection factors (primary prevention) and structure interventions (secondary and tertiary prevention)^16,26,46^.

## Conclusions, limitations and future directions

The results of this study indicate that avatars can exert a nudging effect on human behavior when performing tasks in a virtual environment by affecting—both positively and negatively—one’s tendency to take risks. They also offer an initial empirical assessment of the actual effect of virtual assistance on human behavior by providing a basis for critically evaluating the introduction of these agents in sensitive virtual contexts involving adolescents such as gaming, that can actually lead to maladaptive behavior^28^.

The findings of this study open to a range of directions to be taken for future investigations as also cued by the various studies investigating risk taking in adolescence under the influence of peers, thus adding information on the phenomenon. For example, examining the influence of the mere presence of a virtual avatar in a silent condition is one of such options. Studies on peer pressure draw us a rather complex picture in this respect^54,56,57^. Sommerville and colleagues^54^, for example, found that the influence of mere peer presence may exert different effects on behavior depending on conditions. Negative effects on risky behavior were in fact found as a function of variables such as peer monitoring, reputation management, social benefits and excitement, and arousal increase; opposite, mitigating effects on behavior were found with age increase, in cold decision contexts, and when peers were present, though without actively monitoring behavior.

Additionally, the avatars used in this study represented a human young adult figure and a robot. Although no differences were found between solicitations made by the two, a further interesting opportunity to enrich the present data would be to specifically evaluate the influence on ado’s behavior exerted by virtual agents representing humans of different ages^11–13^. Ruggeri et al.^58^, for example, tested the effects of a peer versus an adult model on children’s and adolescents’ prosocial behavior and showed that children were more likely to follow the recommendations of the adult model whereas adolescents were more likely to follow the recommendations of the peer model. Thus, the findings in this study might have underestimated the effect of peer nudging by designing an avatar with more adult-like features^58^. Furthermore, socio-demographic factors could be included in the design, such as socioeconomic status, ethnicity, family structure, school environment, neighborhood characteristics, and so on. These variables represent important as well as very interesting factors that can actually target and characterize adolescents’ risk-taking behavior in a very specific manner.

Some limitations should be also acknowledged, that prevent us from generalizing our results to the entire spectrum of adolescents. First, our sample was mostly composed of male students. As previous work has shown that boys generally tend to exhibit greater risk-taking behavior than girls, future research should outbalance male and female participants, possibly in a wider age range, to properly address both gender and age issues discussed above. Alongside age and gender, future studies should also evaluate the effect of cognitive processes (e.g., the ability to estimate the probabilities of consequences of actions and executive function skills), and situational influences (e.g., parental monitoring)^59^ on adolescents’ behavior when operating in virtual environments.

In addition, in this study, the playing-alone condition was always presented first, followed by the avatar conditions (incitement or discouragement). This was done so as not to spoil the playing-alone condition—which is thought to assess individual risk-taking tendency—with the experimental tasks having potential carryover effects. However, we note that the playing-alone condition could conceivably drag effects due to the familiarization phase with the BART task itself, which would lead to a plausible increase in the scores of the dependent variables. As an alternative to the mixed design used in this study, a full between-subjects design could perhaps bring out further differences there were here undervalued.

Finally, while in the present study we focused on avatars acting in a virtual environment, it would be interesting to further evaluate the effects of these agents by contrasting their influence against that evoked by a real human or a real robot agent. It would then be noteworthy to also evaluate differences in attribution of mental states to the avatars with respect to the human to better outline the actual level of anthropomorphizing^60–63^. This would delineate the specificity of the avatars’ effects found here.

## Methods

### Participants

One-hundred and fifteen (115) Italian adolescents (34 girls, *mean age* = 17 years, *SD* = 0.65; 81 boys, *mean age* = 17.36 years, *SD* = 0.98) took part in the study (estimated sample size with G*Power tool: linear multiple regression, fixed model: effect size f = 0.15; alpha prob err = 0.05; n. predictors = 8; power = 0.95, N = 89). The study was carried out in secondary schools. The students in 4^th^ and 5^th^ grade were all invited to participate in the study during the regular school activity. Two participants were excluded from data analysis as extreme cases in at least 50% of the conditions, as detailed in the results above. Participants were informed about the experimental procedure, the measurement items, and the materials. Informed consent was obtained from all participants and/or their legal guardian(s) in line with the Declaration of Helsinki and its revisions, as well as in accordance with the requirements of the ethics committee, *Committee of the Department of Psychology (CERPS), Università Cattolica del Sacro Cuore, Milan*, which approved the study.

### General procedure

The study was carried out in school in a dedicated pc room where each participant could link online to the experiment. Administration days were agreed upon with the schoolteachers and involved a whole class at a time.

Following the link, the study opened with instructions and a request to self-generate the identification code and record age, gender, nationality, and comprehension of the Italian language (control). This section was followed by two BART tasks presented in a fixed sequence: one played alone, the other with an avatar, as described below. Upon completion of the BART, the participants were administered the Barratt Impulsiveness scale (BIS), assessing one’s tendency to impulsive behavior and validated in Italian^64^.

### Balloon analogic risk taking (BART) task

#### General logic

The BART tasks were programmed following the general logic of the original task^43^ according to which participants have to inflate a virtual balloon on a computer screen. Participants play 30 rounds of the BART (i.e., pumped up 30 balloons) in each condition (playing-alone, playing with the avatar), in line with previous research using the BART. The more the balloon is inflated, the higher the score obtained. However, the balloon can explode at any time on a probabilistic base. The participant’s risk-taking is weighted against the probability of gain. A detailed description of BART is in Supplemental Material.

**Figure 2:**
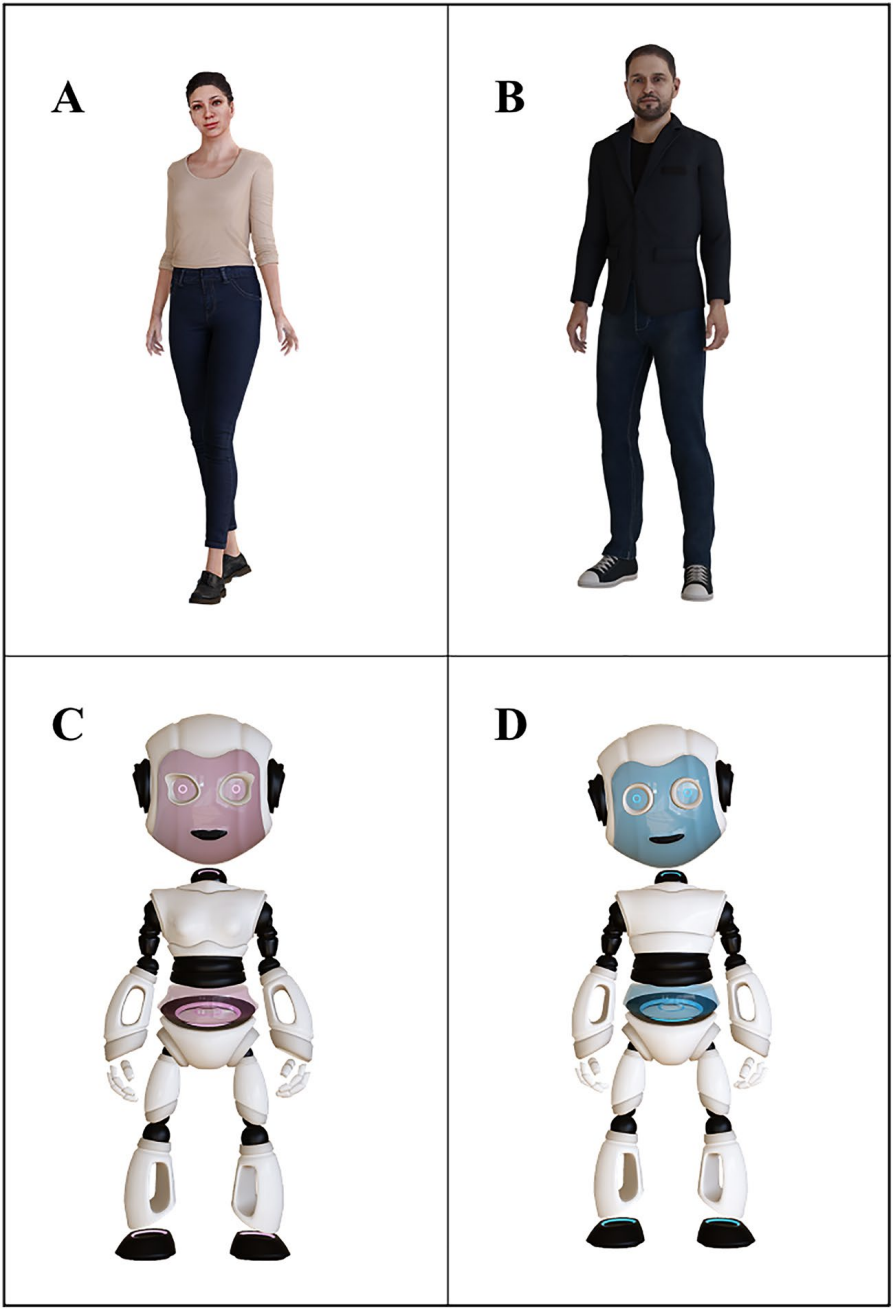
(**A-D**) Images of the avatars used in the study: (**A,B**) represent the human avatar, male and female respectively; (**C,D**) represent the robotic avatar, male and female respectively. The design and programming of the avatars was by QuestIT s.r.l. (https://www.quest-it.com/).

The participants played two BART games: (1) alone, and (2) with one of four avatars (human–HA; and robot–RA), each male or female, in either an incitement or discouragement condition. The HA and RA had the same voice but changed in the level of anthropomorphism. Figure 2 depicts the avatars selected for this study and for which no consent to publish is needed as the images do not represent real human faces. The programming of the BART games and the design and programming of the avatars was by QuestIT s.r.l. (https://www.quest-it.com/).

#### BART 2: playing with the avatar

The task opened with the avatar introducing itself (see Supplemental Material). The avatar began with incitement or discouragement phrases (depending on the condition) from the start and kept inciting or discouraging with a randomized interval between 3 and 7 s. The incitement and discouragement sentences are provided in Supplemental Material.

#### Experimental design

The study is a mixed design. Each participant was administered 2 BARTs as follows: (1) playing alone; (2) playing with an avatar (within-subjects factor). Each participant played with either a human or robot avatar, and only in the incitement or discouragement condition. The program was set to balance the number of participants within each condition (see descriptive data below, and Supplemental Material for details).

#### Dependent variables

Risk taking was evaluated as a function of the following indexes calculated within each BART (alone and with avatar): (1) total pumps in all rounds, i.e., calculated at the end of each BART (*pumps*); total score at the end of each BART (*gain*); total number of trials resulted in an explosion calculated at the end of each BART (*explosion*).

### BIS11

The Barratt Impulsiveness Scale (BIS-11^65^) is a widely used questionnaire to assess the construct of impulsivity^66^, see also^67^. The current version consists of 30 items describing common impulsive or non-impulsive behaviors and it consists of three dimensions: (1) motor impulsivity, defined as the tendency to act without thinking, on the spur of the moment (motor-activation); (2) cognitive impulsivity, understood as the tendency to make quick decisions, and lack of concentration on the task (attention); 3) unplanned impulsivity, underpinning behavior characterized by poor evaluation of consequences (lack-of-planning). The items are rated on a 4-point scale: rarely/never = 1; occasionally = 2; often = 3; almost always/always = 4. The total score is calculated by summing the scores on each item.

### Data analysis

Firstly, we evaluated whether all BART variables were associated with each other (Pearson’s r), thus reflecting the construct of risk-taking, and predicted a positive correlation between the three indexes. Secondly, correlation analyses (Pearson’s r) were carried out between risk-taking behavior when playing alone and in experimental conditions (incitement and discouragement) to evaluate whether participants played the two BARTs in a consistent way—i.e., adopting similar intra-subject behavioral patterns. Specifically, those who tended to play in a riskier manner did so also in the conditions with the avatars. Then, we evaluated differences between conditions (playing-alone, playing with avatar), modality (incitement, discouragement), and type of avatar (HA, RA) on each BART variable (*pumps, gain, explosions*) also as a function of participants’ gender. Independent 2 × 2x2 × 2 general linear model analyses (GLM) were therefore carried out for each dependent variable modeling 2 levels of condition as the within-subject variable, and 2 levels of modality, 2 levels of type of avatar, and 2 levels of gender as the between-subject variables. Finally, three independent regression analyses evaluated the predictive effect of the BART variables. More specifically, we regressed *pumps, gain, and explosions*, separately, on the independent variables *modality* (incitement, discouragement) and *type of avatar* (HA, RA) controlling for participants’ age and gender (model 1); impulsive behavior (BIS11; model 2); and BART scores when playing alone (model 3). This last pass was important to evaluate the effect of avatars’ incitement and discouragement on risky behavior after controlling for individual risk-taking tendencies assessed independent of nudging (playing-alone condition).

## Supplementary Information

Supplementary Information.

## Data Availability

The datasets generated during and/or analyzed during the current study are available from the corresponding author on reasonable request.
