# Author Correction: Virtual agents and risk-taking behavior in adolescence: the twofold nature of nudging

**DOI:** 10.1038/s41598-023-38995-w

**Published:** 2023-07-20

**Authors:** Cinzia Di Dio, Federico Manzi, Laura Miraglia, Michaela Gummerum, Simone Bigozzi, Davide Massaro, Antonella Marchetti

**Affiliations:** 1 Research Unit On Theory of Mind, Department of Psychology, Università Cattolica del Sacro Cuore, Milan, Italy; 2 Research Unit On Robopsychology in the Lifespan, Department of Psychology, Università Cattolica del Sacro Cuore, Milan, Italy; 3 Department of Psychology, University of Warwick, Coventry, UK; 4 QuestIT s.r.l., Siena, Italy

Correction to: *Scientific Reports* https://doi.org/10.1038/s41598-023-38399-w, published online 11 July 2023

The original version of this Article contained an error in the name of author Davide Massaro, which was incorrectly given as Massaro Davide.

The original Article has been corrected.

