# Supplementary material for: Virtual agents and risk-taking behavior in adolescence: the twofold nature of nudging
Source: Sci Rep. 2023 Jul 11;13:11242. doi: 10.1038/s41598-023-38399-w (PMC10336118; doi:10.1038/s41598-023-38399-w)
Supplement: Supplementary file 1 — Supplementary Information. [file 41598_2023_38399_MOESM1_ESM.pdf]

Supplemental Material

Method

BART

Rationale

Each BART consisted of 30 trials (rounds) in which participants were asked to press the "inflate" button to inflate a balloon displayed on the computer monitor. In total, participants had to inflate 30 different balloons within each BART, i.e., 60 total (30 when playing alone; 30 when playing with the avatars). Each time the “inflate” key/button was pressed, the balloon was inflated by 1 point that was added to the "temporary score" shown on the screen. Next to this, there was also an indication of the "total score" which was recalculated at each round. The color of the balloon randomly changed in each round to aid attention. Also, each pump corresponded to an increase in the volume of the balloon to foster the perception of a causal effect between behaviour and balloon inflation.

Burst logic: the balloon randomly bursted after each pump with a probability of 1/128 - as the pumps progressed, the probability of bursting increased (e.g., 1/127, 1/126 ...). Each round ended when: a. the participant clicked on the "cash out" button, or b. the balloon exploded.

At the end of the first BART, a screen appeared with the total score.

Condition with the avatars

The task opened with a page where the agent (virtual human, robot) introduced itself as follows: “hello, I am your partner in this second part of the game. The game is exactly the same as the one you’ve just played. Be careful! (discouragement condition) / be brave! (encouragement condition). When you are ready, press enter”.

Encouragement phrases (presented randomly within each round)

1. go ahead
2. keep pressing
3. don't stop
4. don't be afraid
5. keep inflating
6. inflate
7. come on
8. go on
9. go ahead
10. don't stop
11. press
12. go, go

Phrases of discouragement

1. Attention
2. Take it easy
3. Do not overdo it
4. Stop
5. Caution
6. Be careful
7. Stop
8. Be careful to inflate
9. Be cautious
10. Watch out

11. Easy, easy
12. Wait

Experimental Design
The study was a 2x2x2x2 mixed design, with 2 levels of condition (alone, with agent) as within-subject factors, and 2 levels of encouragement type (positive, negative), 2 levels of agent (virtualized human and robot) and 2 levels of agent's gender (male, female) as between-subject factors.

Thus, each participant was administered 2 BARTs as follows: 1) playing alone; 2) playing with one of the two agents (human, or robot – either male or female) and only in an encouragement/discouragement condition. Therefore, each participant randomly played in one of 8 conditions as follows:

1. BART alone + BART with robot (M) in encouragement condition
2. BART alone + BART with robot (M) in discouragement condition
3. BART alone + BART with virtual agent (M) in encouragement condition
4. BART alone + BART with virtual agent (M) in discouragement condition
5. BART alone + BART with robot (F) in encouragement condition
6. BART alone + BART with robot (F) in discouragement condition
7. BART alone + BART with virtual (F) agent in encouragement condition
8. BART alone + BART with virtual (F) agent in discouragement condition

The program was set to balance the number of participants within each condition (see descriptive data in results).

DEBRIEFING

In the title of the study in the information form, it was deliberately omitted any reference to the word *risk*. This was to avoid any bias in the participants' response to the BART tasks. However, at the end of the study, there was a written debriefing explaining more specifically the aim of the study (see detailed description below).

Debriefing after the study

At the end of the study, the participant were released with written debriefing regarding the specific purpose of the study as follows:

"The study has ended. We thank you for participating.

To further clarify the purpose of this study, please be informed that it aims to assess the risk propensity and the effect of an AI-based avatar on the behaviour of young boys/girls when operating online. More specifically, in the balloon game, each participant underwent different experimental conditions, i.e. situations in which the artificial playmate could be a virtual robot (male or female) or a virtual person (male or female). These avatars gave indications in some cases to be more risky, in others to be more cautious. Each of these conditions was used to assess whether the participant's behaviour changes when playing alone (in the first part of the game) and when playing with a virtual mate, and thus measure whether playing with the virtual mate increases (risk encouraging conditions) or decreases (risk discouraging conditions) the participant's tendency to risk.

We omitted this information at the beginning of your participation in order not to influence your spontaneous behaviour at the BART game. We reiterate that your performance at the game as well

as the data provided to us remain anonymous, as specified in the information you had read before participating in the study.

Results

Supplementary Table 1

Regression analysis for variables predicting performance at the BART task played in the experimental condition (with avatar).

Predictor regression 1: pumps (N = 113)

Predictor regression 2: gain (N = 113)

Predictor regression 3: explosions (N = 113)

**Model**	**Independent Variables**	**Dependent Variables**								
		Pumps			*Gain*			*Explosions*		
		B	SE(B)	*β*	B	SE(B)	*β*	B	SE(B)	*β*
1	*Age range*	2.182	38.268	.006	37.003	25.621	0.139	0.251	0.427	0.057
	Gender	19.131	76.580	.024	-3.459	51.27	-0.006	0.441	0.855	0.05
2	*Age range*	-3.371	38.426	-.009	33.309	25.782	0.125	0.192	0.43	0.044
	Gender	16.884	77.163	.021	-9.692	51.773	-0.018	0.334	0.864	0.038
	BIS11-Att	11.324	13.305	.097	5.564	8.927	0.07	0.085	0.149	0.065
	BIS11-Mot	8.968	8.673	.117	2.973	5.819	0.057	0.041	0.097	0.048
	BIS11-NonPl	.242	7.898	.003	5.513	5.299	0.104	0.098	0.088	0.111
3	*Age range*	-30.325	32.360	-.077	19.032	21.04	0.071	0.09	0.328	0.02
	Gender	-31.065	64.879	-.039	-55.49	42.52	-0.104	-0.061	0.66	-0.007
	BIS11-Att	3.770	11.176	.032	2.46	7.267	0.031	0.013	0.114	0.01
	BIS11-Mot	4.305	7.282	.056	-2.769	4.791	-0.053	-0.097	0.076	-0.113
	BIS11-NonPl	1.065	6.603	.014	6.647	4.309	0.126	0.087	0.067	0.1
	Play-alone cond	.578	.084	.565**	0.475	0.063	0.6**	0.538	0.061	0.674**
4	*Age range*	-40.927	32.055	-.104	12.069	20.657	0.045	0.006	0.324	0.001
	Gender	-25.138	63.676	-.032	-50.432	41.372	-0.094	-0.002	0.646	0
	BIS11-Att	.432	11.058	.004	-0.181	7.126	-0.002	-0.025	0.112	-0.019
	BIS11-Mot	5.547	7.172	.072	-1.579	4.68	-0.03	-0.077	0.074	-0.089
	BIS11-NonPl	.104	6.487	.001	5.91	4.197	0.112	0.078	0.066	0.089
	Play-alone cond	.570	.083	.557**	0.461	0.062	0.583**	0.525	0.06	0.657**
	Agent type	-16.996	56.178	-.024	10.468	36.312	0.022	0.519	0.572	0.064
	Modality	141.467	57.163	.197*	105.22	37.037	0.217*	1.433	0.585	0.178*

*p < .01; **p < .001.
